# Trends and determinants of prevalence, awareness, treatment and control of dyslipidaemia in canton of Geneva, 2005–2019: Potent statins are underused

**DOI:** 10.1016/j.ijcrp.2023.200187

**Published:** 2023-05-19

**Authors:** Pedro Marques-Vidal, Valeriya Chekanova, Carlos de Mestral, Idris Guessous, Silvia Stringhini

**Affiliations:** a Department of Medicine, Internal Medicine, Lausanne University Hospital and University of Lausanne, Lausanne, Switzerland; b National Medical Research Center of Cardiology, Moscow, Russia; c Population Epidemiology Unit, Primary Care Division, Geneva University Hospital, Geneva, Switzerland

**Keywords:** Dyslipidaemia, Epidemiology, Evolution, Treatment, Control

## Abstract

We assessed 1) trends in prevalence, awareness, treatment and control rates of dyslipidaemia and associated factors, 2) the effect of statin generation/potency on control levels and 3) the effect of ESC lipid guidelines, on lipid management. Data from multiple cross-sectional, population-based surveys conducted between 2005 and 2019 in the canton of Geneva, Switzerland, were used. Prevalence, awareness, treatment and control rates of dyslipidaemia were 46.0% and 34.9% (p < 0.001), 67.0% and 77.3% (p = 0.124), 40.0% and 19.9% (p < 0.001), and 68.0% and 84.0% (p = 0.255), in 2005 and 2019, respectively. After multivariable adjustment, only the decrease in treatment rates was significant. Increasing age, higher BMI, history of hypertension or diabetes were positively associated with prevalence, while female sex was negatively associated. Female sex, history of diabetes or CVD were positively associated with awareness, while increasing age was negatively associated. Increasing age, smoking, higher BMI, history of hypertension, diabetes or CVD were positively associated with treatment, while female sex was negatively associated. Female sex was positively associated with control, while increasing age was negatively associated. Highly potent statins increased from 50.0% to 87.5% and third generation statins from 0% to 47.5% in 2009 and 2015, respectively. Increased statin potency was borderline (p = 0.059) associated with dyslipidaemia control. ESC guidelines had no effect regarding the prescription of more potent or higher generation statins. We conclude that in the canton of Geneva, treatment of diagnosed dyslipidaemia is low, but control is adequate. Women are undertreated but better controlled than men. The most potent hypolipidemic drugs are underused.

## 1 Introduction

Cardiovascular disease (CVD) is responsible for over half of all deaths in Europe [1]. Numerous randomized controlled trials have shown that adequate management of dyslipidaemia (high LDL cholesterol levels) translates into a reduction in fatal and non-fatal CVD [2]. This has led the issuing and updating of guidelines for the management of dyslipidaemia in 2020 in Europe [3] and in 2019 in the USA [4].

It remains unknown if the issuing of guidelines affects the management of this condition in the general European population, and most studies on this topic have been conducted in the USA. One study reported that in the first year after the ACC/AHA guideline introduction in late 2013, more patients were prescribed a statin, but it was unclear whether the new guidelines were strictly adhered to regarding intensity of statin therapy [5]. Another study showed that many primary care clinicians fail to implement guidelines in their clinical practice [6], and another showed that, while the majority of clinicians report adoption of the guideline recommendations, observed lipid management decisions are frequently discordant [7]. A German study showed a decrease in LDL-C levels after the issuing of the 2011 guidelines, but this decrease was no longer present 4 years afterwards, and the rate of patients meeting recommended LDL-targets decreased over time [8]. In a previous study conducted in Switzerland, we showed that the increase over time in dyslipidaemia prevalence was not paralleled by a similar increase in lipid-lowering drugs [9]. As the analyses were conducted in a limited period, it was not possible to assess whether the publication of guidelines influenced dyslipidaemia management.

Therefore, the aims of the present study were to assess 1) the trends in prevalence and management of dyslipidaemia, 2) the trends in lipid levels and 3) the effect of the year of publication of the ESC lipid guidelines, on management of dyslipidaemia and on lipid levels in a large representative sample of adults living in the state of Geneva, Switzerland.

## 2 Methods

### 2.1 Population and sampling

We used data from the Bus Santé study, an ongoing yearly population-based cross-sectional study of adults living in the state of Geneva, Switzerland. Briefly, yearly health examination surveys were conducted in ∼1000 men and women, drawn from independent samples of residents since 1992 [10]. Participant selection was based on a residents list provided by the local government, including individuals aged 35–74 years until 2017. This list includes the first and last name, sex, age, nationality, and address of each resident of Geneva. Random sampling in age–sex-specific strata was proportional to the corresponding frequencies in the population. In the first letter mailed to a potential subject, the selected individual was asked to indicate the day and time that would be convenient to come to the mobile unit. In the case of nonresponse, up to seven phone attempts were made to reach the person at different times of the day and various days of the week, including Saturday and Sunday. Two more mailings were sent when a selected individual could not be reached by phone. A person who had not been reached after three mailings and seven phone calls was replaced using the same selection protocol. The recruitment of a potential subject lasted from 2 weeks to 2 months. Each participant was assessed only once, as each year a new random sample is selected.

### 2.2 Ethical statement

The Bus Santé study was approved by the local institutional review board (Commission Cantonale d’Ethique de la Recherche de Genève; IRB00003116). All participants provided written informed consent.

### 2.3 Prevalence, awareness, treatment and control of dyslipidaemia

Participants completed a self-administered, standardized questionnaire covering lifestyle factors, reproductive history, and classic CVD risk factors. Awareness of dyslipidaemia was considered when the participant responded positively to the question “have you ever been told you have too much blood cholesterol?”. Treatment of dyslipidaemia was considered if the participant responded positively to the question “Are you currently taking medicine for high blood cholesterol?”. Participants were invited to provide the names of the lipid-lowering medicines they took. Lipid-lowering drugs were further classified into statins, fibrates, and other lipid-lowering drugs based on the Anatomical Therapeutic Chemical (ATC) classification system of the WHO. For statins, a further classification regarding the generation and potency was performed in the first and second follow-ups. As no information was available regarding the posology, it was not possible to classify statins accordingly, and classification was performed as suggested in other studies [11,12] (Supplementary Table 1). Finally, single pills containing combinations of lipid-lowering drugs (i.e. statins + fibrates or statins + ezetimibe) were identified.

Plasma total, LDL and HDL-cholesterol and triglycerides were assessed on fasting subjects. As there is no consensus regarding which lipid guidelines to use in Switzerland, the European Society of Cardiology (ESC) guidelines of 2016 were used [13] and the SCORE equation calibrated for Switzerland was applied [14]. Similarly to a previous study [15], participants were classified as presenting with dyslipidaemia if 1) their LDL-cholesterol levels exceeded the threshold for the corresponding SCORE risk category; 2) they responded positively to the question “did somebody ever tell you that you had too much blood cholesterol?” or 3) they reported taking lipid-lowering drugs. Participants were considered as controlled if their LDL-cholesterol levels were below the threshold indicated in the guidelines (Supplementary Table 2). The following rates were used: prevalence of dyslipidaemia, aware participants (numerator) among participants with dyslipidaemia (denominator), treated participants (numerator) among aware participants (denominator) and controlled participants (numerator) among treated participants (denominator).

### 2.4 Covariates

Nationality was defined as Swiss and non-Swiss. Marital status was categorized as single, married or living in couple, divorced, and widowed. Smoking was categorized into never, former (irrespective of the time since quitting) and current. Educational level was categorized as primary, secondary, and tertiary education. Personal history of CVD was considered if the participant responded positively to any of the questions “have you ever been told that you had myocardial infarction/angina pectoris/closure of a brain or leg artery?”.

Participants also underwent a physical examination and blood testing. Height and weight were measured using standard procedures; body mass index (BMI) was computed and categorized into low-normal (<25 kg m^−2^), overweight (25≤. <30 kg m^−2^) and obese (≥30 kg m^−2^).

Hypertension was considered if participants reported taking antihypertensive drugs or if they had a systolic blood pressure ≥140 mm Hg or if they had a diastolic blood pressure ≥90 mm Hg. Diabetes was considered if participants reported taking antidiabetic drugs or if their fasting plasma glucose was >7 mmol/L.

### 2.5 Exclusion criteria

Participants were excluded if 1) they had missing data for lipids or for computation of SCORE or 2) they had missing data for any covariate.

### 2.6 Statistical analyses

Data were analysed using Stata V.16.1 (Stata Corp, College Station, TX, USA). Descriptive statistics were presented as number of participants (percentage) for categorical variables and as average ± standard deviation for continuous variables. Bivariate analyses were performed using chi-square for categorical variables and student's t-test for continuous variables.

Both crude and age-standardized rates were computed. Direct standardization was performed using the standard EU population for 2013 [16]. Multivariable analysis of crude data was performed using Poisson regression adjusting for age (continuous), gender (man, woman), nationality (Swiss/non-Swiss), marital status (4 categories), smoking categories (never, former, current), BMI categories (normal, overweight, obese), hypertension (yes/no), diabetes (yes/no) and personal history of CVD (yes/no). Results were expressed as incidence-rate ratios, equivalent to odds ratio, and (95% confidence interval).

Trends in lipid levels were assessed by analysis of variance (ANOVA) adjusting for the same covariates as before, plus presence of lipid-lowering drug treatment. Multivariable-adjusted means and corresponding standard errors were computed for each year and a test for trend was performed.

The effect of guidelines was assessed by comparing treatment rates and lipid levels before and after the year immediately following their publication. Two ESC guidelines on dyslipidaemia published in 2011 [17] and 2016 [13] and the AHA guideline published in 2013 [12] were considered, and a dichotomous proxy variable (before/after) was built. This strategy was chosen because the publication of the guidelines in Swiss medical journals occurred approximately one year after the original publication [18], and to have enough time for a guideline to be implemented. Treatment rates were compared using Poisson regression adjusting for the covariates indicated above, while lipid levels were assessed using ANOVA adjusting for the same covariates and including an interaction between year and the proxy. For lipid levels, if an interaction was found, the slopes and corresponding 95% confidence between lipid levels and year were computed before and after the guidelines, adjusting for the same covariates.

For all analyses, statistical significance was set for a two-sided test with P < 0.05.

## 3 Results

### 3.1 Selection of participants

Of the initial 13,123 participants eligible for the study, 1311 (10.0%) were excluded due to lack of lipid data or information to compute SCORE, and a further 517 (3.9%) due to lack of covariates, leaving 11,295 participants (86.1%) for analysis. The comparison between excluded and included participants is summarized in Supplementary Table 3. Excluded participants were significantly younger, more frequently Swiss, single, more frequently of secondary education, never smokers and with normal weight; excluded participants reported less frequently hypertension, diabetes or history of CVD.

### 3.2 Trends in reported awareness, treatment and control rates

**Fig. 1:**
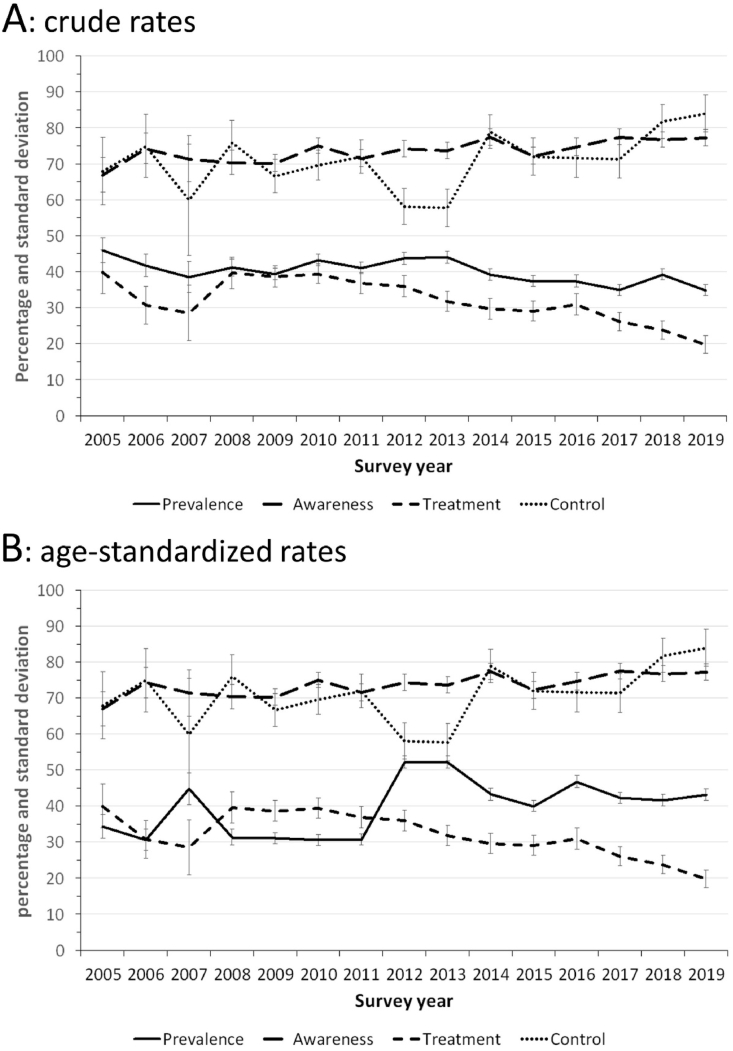
Trends in prevalence, awareness, treatment, and control rates for dyslipidaemia, 2005–2019, Bus Santé study, Geneva, Switzerland. Results are expressed as percentage of participants with dyslipidaemia, aware participants among participants with dyslipidaemia, treated participants among aware, and controlled participants among treated. Panel A: crude rates: panel B: age-standardized rates.

The trends in prevalence, awareness, treatment and control rates are summarized in Fig. 1 panel A for crude rates and panel B for standardized rates. Between 2005 and 2019, prevalence, awareness, treatment and control rates changed from 46.0% to 34.9% (p for trend <0.001), 67.0%–77.3% (p for trend = 0.124), 40.0%–19.9% (p for trend <0.001) and 68.0%–84.0% (p for trend = 0.255), respectively (Fig. 1, panel A). The standardized prevalence, awareness, treatment and control rates evolved from 34.4% to 43.2% (p for trend = 0.047), 53.0%–59.9% (p for trend = 0.017), 22.7%–11.5% (p for trend = 0.040) and 44.0%–50.1% (p for trend = 0.152), respectively (Fig. 1, panel B).

The overall characteristics of the participants with dyslipidaemia, participants aware of their status, treated and controlled are summarized in Table 1. Dyslipidaemia tended to be more frequent among men, older participants, Swiss nationals, participants with a primary or secondary educational level, and former smokers; additionally, dyslipidaemia was more frequent among participants with overweight or obesity, with hypertension, diabetes, or a history of CVD. Awareness of having dyslipidaemia was more frequent among women, younger participants, those with a tertiary education, non-Swiss nationals, as well as participants with diabetes, a history of CVD, and those without hypertension. Treatment of dyslipidaemia was more frequent among men, older participants, those with a primary or secondary educational level, Swiss nationals, former smokers, and participants with overweight or obesity, hypertension, diabetes, and a history of CVD. Control of dyslipidaemia was more frequent among women, younger participants, and those without hypertension (Table 1).

**Table 1:** Bivariate analysis of the characteristics of participants with dyslipidaemia, aware of their status, reported being treated for their status, and with controlled lipid levels, Bus Santé study, Geneva, Switzerland, 2005–2019.

	Dyslipidaemia			Awareness of dyslipidaemia			Treatment among aware			Control among treated		
	No	Yes	P	No	Yes	P	No	Yes	P	No	Yes	P
N	6816	4479		1155	3322		2272	1052		303	718	
Woman (%)	3951 (58.0)	1896 (42.3)	<0.001	420 (36.4)	1475 (44.4)	<0.001	1107 (48.7)	371 (35.3)	<0.001	87 (28.7)	273 (38.0)	0.004
Age (years)	46.3 ± 9.8	57.3 ± 11.9	<0.001	62.9 ± 9.4	55.3 ± 12.0	<0.001	52.0 ± 11.8	62.4 ± 8.8	<0.001	68.3 ± 7.2	60.0 ± 8.3	<0.001
Educational level (%)			<0.001			0.032			<0.001			0.868
Primary	497 (7.3)	443 (9.9)		103 (8.9)	340 (10.2)		208 (9.2)	134 (12.7)		40 (13.2)	90 (12.5)	
Secondary	2823 (41.4)	2096 (46.8)		578 (50)	1517 (45.7)		965 (42.5)	550 (52.3)		160 (52.8)	372 (51.8)	
Tertiary	3496 (51.3)	1940 (43.3)		474 (41)	1465 (44.1)		1099 (48.4)	368 (35.0)		103 (34.0)	256 (35.7)	
Swiss nationality (%)	4290 (62.9)	3180 (71.0)		871 (75.4)	2307 (69.5)		1553 (68.4)	757 (72.0)	0.036	230 (75.9)	505 (70.3)	0.070
Marital status (%)			<0.001			0.002						0.547
Single	925 (13.6)	451 (10.1)		87 (7.5)	364 (11.0)		286 (12.6)	79 (7.5)		23 (7.6)	53 (7.4)	
Married/couple	4635 (68.0)	3082 (68.8)		803 (69.5)	2277 (68.5)		1506 (66.3)	771 (73.3)		229 (75.6)	523 (72.8)	
Divorced	939 (13.8)	681 (15.2)		181 (15.7)	500 (15.1)		358 (15.8)	144 (13.7)		34 (11.2)	105 (14.6)	
Widowed	317 (4.7)	265 (5.9)		84 (7.3)	181 (5.5)		122 (5.4)	58 (5.5)		17 (5.6)	37 (5.2)	
Smoking status (%)			<0.001			0.253			<0.001			0.429
Never	3444 (50.5)	1970 (44.0)		532 (46.1)	1437 (43.3)		1041 (45.8)	402 (38.2)		117 (38.6)	271 (37.7)	
Former	1915 (28.1)	1601 (35.7)		399 (34.6)	1202 (36.2)		764 (33.6)	437 (41.5)		118 (38.9)	307 (42.8)	
Current	1457 (21.4)	908 (20.3)		224 (19.4)	683 (20.6)		467 (20.6)	213 (20.3)		68 (22.4)	140 (19.5)	
BMI (kg/m^2^)	24.6 ± 4.3	26.2 ± 4.7	<0.001	26.2 ± 3.9	26.3 ± 5.0	0.683	25.6 ± 5.0	27.7 ± 4.6	<0.001	27.6 ± 4.5	27.6 ± 4.6	0.969
BMI categories (%)			<0.001			<0.001			<0.001			0.505
Normal	4136 (60.7)	1882 (42.0)		463 (40.1)	1418 (42.7)		1108 (48.8)	312 (29.7)		87 (28.7)	217 (30.2)	
Overweight	1965 (28.8)	1795 (40.1)		519 (44.9)	1275 (38.4)		829 (36.5)	448 (42.6)		138 (45.5)	299 (41.6)	
Obese	715 (10.5)	802 (17.9)		173 (15)	629 (18.9)		335 (14.7)	292 (27.8)		78 (25.7)	202 (28.1)	
Hypertension (%)	897 (13.2)	1772 (39.6)	<0.001	575 (49.8)	1197 (36.0)	<0.001	564 (24.8)	630 (59.9)	<0.001	229 (75.6)	385 (53.6)	<0.001
Diabetes (%)	270 (4.0)	558 (12.5)	<0.001	81 (7)	477 (14.4)	<0.001	207 (9.1)	270 (25.7)	<0.001	70 (23.1)	186 (25.9)	0.345
History of CVD (%)	145 (2.1)	356 (8.0)	<0.001	47 (4.1)	309 (9.3)	<0.001	77 (3.4)	234 (22.2)	<0.001	71 (23.4)	160 (22.3)	0.689

BMI, body mass index. Results are expressed as number of participants (column percentage) for categorical variables or as average ± standard deviation for continuous variables. Between-group comparisons performed using chi-square for categorical variables and student's t-test for continuous variables.

The results of the multivariable analysis of the factors associated with prevalence, awareness, treatment and control of dyslipidaemia are summarized in Table 2. Increasing age, increasing BMI, and history of hypertension or diabetes were positively associated with prevalence of dyslipidaemia, while female sex was negatively associated. Female sex and history of diabetes or CVD were positively associated with awareness, while increasing age was negatively associated. Increasing age, current smoking, increasing BMI, and history of hypertension, diabetes or CVD were positively associated with treatment, while female sex and increasing education were negatively associated. Female sex was positively associated with control, while increasing age was negatively associated (Table 2).

**Table 2:** Multivariable analysis of the characteristics of participants with dyslipidaemia, aware of their status, reported being treated for their status, and with controlled lipid levels, Bus Santé study, Geneva, Switzerland, 2005–2019.

	Prevalence	P	Awareness of dyslipidaemia	P	Treatment among aware	P	Control among treated	P
Year (per 1 unit-increase)	1.00 (0.99–1.01)	0.232	1.00 (0.99–1.01)	0.390	0.97 (0.95–0.98)	<0.001	1.02 (1.00–1.04)	0.112
Woman vs. man	0.75 (0.70–0.79)	<0.001	1.12 (1.04–1.20)	0.003	0.79 (0.68–0.90)	0.001	1.24 (1.05–1.47)	0.010
Age (per decade)	1.51 (1.46–1.55)	<0.001	0.86 (0.83–0.89)	<0.001	1.60 (1.50–1.72)	<0.001	0.73 (0.67–0.79)	<0.001
Educational level (%)								
Primary	1 (ref.)		1 (ref.)		1 (ref.)		1 (ref.)	
Secondary	0.91 (0.82–1.02)	0.094	0.98 (0.87–1.11)	0.749	0.96 (0.79–1.16)	0.665	1.08 (0.85–1.37)	0.548
Tertiary	0.90 (0.81–1.01)	0.063	0.98 (0.87–1.11)	0.784	0.80 (0.65–0.99)	0.039	1.10 (0.86–1.41)	0.456
P-value for trend	0.063		0.784		0.039		0.456	
Swiss nationality vs. other	1.03 (0.96–1.10)	0.359	1.00 (0.93–1.09)	0.904	0.97 (0.85–1.12)	0.708	1.01 (0.85–1.20)	0.896
Marital status								
Single	1 (ref.)		1 (ref.)		1 (ref.)		1 (ref.)	
Married/couple	0.91 (0.83–1.01)	0.078	1.02 (0.91–1.14)	0.722	1.07 (0.84–1.35)	0.595	1.12 (0.84–1.49)	0.456
Divorced	0.91 (0.81–1.03)	0.132	1.01 (0.88–1.16)	0.851	0.92 (0.69–1.21)	0.534	1.20 (0.86–1.68)	0.282
Widowed	0.97 (0.83–1.13)	0.693	0.96 (0.80–1.15)	0.665	1.10 (0.79–1.55)	0.569	1.08 (0.71–1.65)	0.723
Smoking status								
Never	1 (ref.)		1 (ref.)		1 (ref.)		1 (ref.)	
Former	1.06 (0.99–1.13)	0.104	1.05 (0.98–1.14)	0.180	1.06 (0.93–1.22)	0.374	1.08 (0.92–1.28)	0.342
Current	1.16 (1.07–1.25)	<0.001	0.98 (0.89–1.08)	0.662	1.24 (1.05–1.47)	0.013	0.87 (0.70–1.07)	0.182
P-value for trend	<0.001		0.662		0.013		0.118	
BMI categories (%)								
Normal	1 (ref.)		1 (ref.)		1 (ref.)		1 (ref.)	
Overweight	1.17 (1.09–1.25)	<0.001	0.99 (0.92–1.07)	0.843	1.19 (1.02–1.39)	0.023	0.98 (0.82–1.18)	0.853
Obese	1.17 (1.09–1.25)	<0.001	0.99 (0.92–1.07)	0.843	1.19 (1.02–1.39)	0.023	0.98 (0.82–1.18)	0.853
P-value for trend	<0.001		0.267		<0.001		0.888	
Hypertension (yes vs. no)	1.19 (1.11–1.28)	<0.001	0.95 (0.88–1.03)	0.226	1.36 (1.18–1.57)	<0.001	0.89 (0.75–1.04)	0.141
Diabetes (yes vs. no)	1.16 (1.05–1.27)	0.003	1.24 (1.12–1.38)	<0.001	1.26 (1.09–1.46)	0.002	1.16 (0.98–1.38)	0.093
History of CVD (yes vs. no)	1.09 (0.98–1.22)	0.114	1.29 (1.14–1.46)	<0.001	1.63 (1.40–1.90)	<0.001	1.07 (0.89–1.29)	0.447

BMI, body mass index. Results are expressed as incidence-rate ratios and (95% confidence interval). Multivariate analysis performed using Poisson regression.

### 3.4 Trends in lipid-lowering drugs

**Fig. 2:**
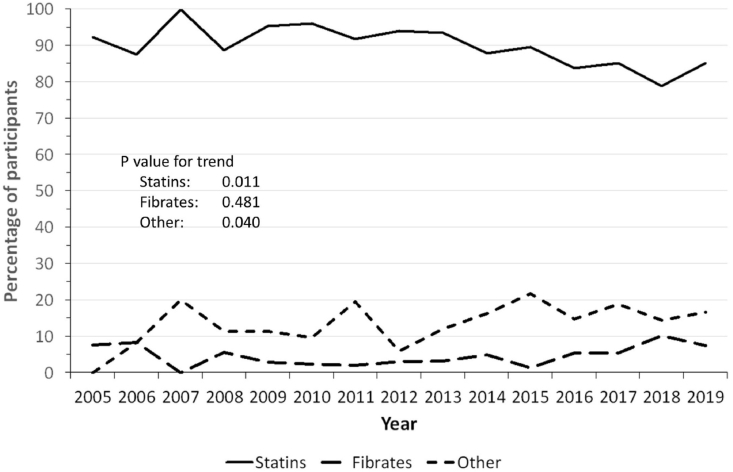
Trends in lipid-lowering drugs prescribed, 2005–2019, Bus Santé study, Geneva, Switzerland. Results are expressed as percentage of treated participants taking the drug.

**Fig. 3:**
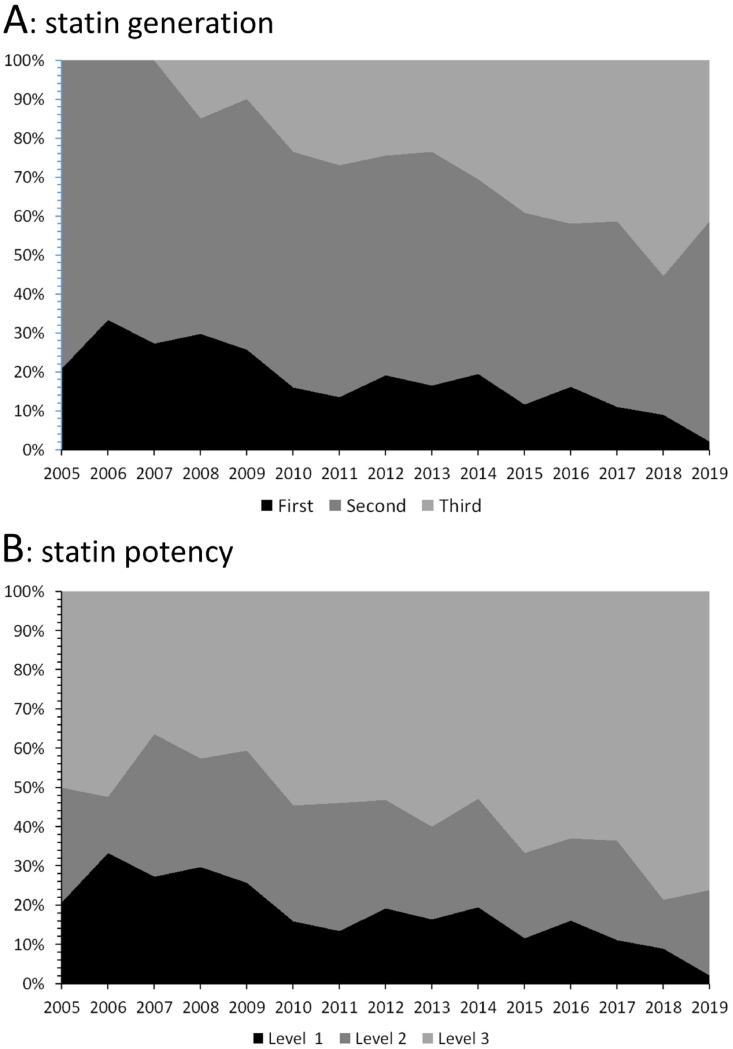
Trends in generation and potency of statins prescribed to participants with dyslipidaemia, 2005–2019, Bus Santé study, Geneva, Switzerland. Results are expressed as percentage. Panel A: statin generation; panel B: statin potency. For the definition of generation and potency, please consult Supplementary Table 1.

Statins were the most prescribed drugs, although their predominance tended to decrease relative to other lipid-lowering drugs and, to a lesser degree, to fibrates (Fig. 2). The trends regarding the generation and potency of prescribed statins are summarized in Fig. 3 panel A for generation and panel B for potency. Between 2005 and 2019, first and second generation statins decreased from 20.8% to 2.2% and from 79.2% to 56.5%, while third generation statins increased from 0% to 41.3% (Fig. 3, panel A). Statins with potency 1 or 2 decreased from 20.8% to 2.2% and from 29.2% to 21.7%, while the most potent statins increased from 50.0% to 76.1% (Fig. 3, panel B). Finally, prevalence of single-pill combinations of lipid-lowering drugs was low, being taken by only 0.6% of participants in 2019.

Multivariable analysis showed that neither statins, nor fibrates, nor other lipid-lowering drugs were associated with control rates (Table 3). Increased statin potency was borderline associated (p = 0.051) with a higher likelihood of controlled dyslipidaemia, while no association was found with statin generation (Table 3).

**Table 3:** Effect of lipid-lowering drug class on dyslipidaemia control, Bus Santé study, Geneva, Switzerland, 2005–2019.

	Model 1	P	Model 2	P	Model 3	P
Fibrate (yes vs. no)	1.31 (0.80–2.13)	0.286	1.28 (0.60–2.72)	0.526	1.28 (0.60–2.74)	0.524
Other (yes vs. no)	1.09 (0.87–1.36)	0.459	1.14 (0.88–1.48)	0.305	1.10 (0.86–1.39)	0.448
Statins (yes vs. no)	1.53 (0.99–2.37)	0.057	–	–		
Statin potency						
Level 1	–		1 (ref.)		–	
Level 2	–		1.15 (0.88–1.51)	0.299	–	
Level 3	–		1.26 (1.00–1.59)	0.050	–	
P-value for trend			0.051			
Statin generation						
First	–		–		1 (ref.)	
Second	–		–		1.22 (0.97–1.54)	0.096
Third	–		–		1.25 (0.97–1.62)	0.090
P-value for trend					0.090	

Model 1 tested the three classes of lipid-lowering drugs (fibrates, statins, other); model 2 tested fibrates, other lipid-lowering drugs and the different levels of statin potency: model 3 tested fibrates, other lipid-lowering drugs and the different statin generations. -, not performed. Results are expressed as incidence-rate ratios and (95% confidence interval). Multivariate analysis performed using Poisson regression adjusting for age (continuous), gender (man, woman), nationality (Swiss/non-Swiss), marital status (4 categories), smoking categories (never, former, current), BMI categories (normal, overweight, obese), hypertension (yes/no), diabetes (yes/no) and personal history of CVD (yes/no).

### 3.6 Trends in lipid levels

Total cholesterol levels decreased from 5.73 ± 0.07 mmol/L (multivariable-adjusted average ± standard error) in 2005 to 5.12 0.03 mmol/L in 2019 (p-value for trend<0.001, Supplementary Fig. 1). The corresponding values were 3.65 ± 0.06 and 3.00 ± 0.03 mmol/L for LDL-cholesterol (p-value for trend<0.001, Supplementary Fig. 1) and 1.41 ± 0.03 and 1.60 ± 0.01 mmol/L for HDL (p-value for trend<0.001, Supplementary Fig. 1).

### 3.7 Effect of guidelines on treatment rates and lipid levels

Multivariable analysis of the treatment rates before and after the issuing of the ESC dyslipidaemia guidelines showed no effect for the guidelines published in 2011 (IRR: 0.89, 95% CI 0.72–1.11, p = 0.318), 2013 (IRR: 1.09, 95% CI 0.87–1.37, p = 0.467) or 2016 (IRR: 0.87, 95% CI 0.70–1.09, p = 0.229). Similar findings were observed for control levels: 0.88 (0.68–1.16), (1.08, 0.81–1.44) and 1.08 (0.83–1.40) for the 2011, 2013 and 2016 guidelines, respectively. No effect of the guidelines was found regarding the prescription of more potent or higher generation statins (not shown).

Regarding lipid levels, an interaction was found between the 2011 ESC guidelines and year regarding total (decrease, p = 0.040), HDL (increase, p < 0.001) and LDL (decrease, p < 0.001) cholesterol. Similar results were found for the 2013 AHA guidelines regarding total (p < 0.001) and LDL cholesterol (p < 0.001), but not for HDL cholesterol (p = 0.231), and for the 2016 ESC guidelines regarding total (p < 0.001) and LDL (p = 0.027) cholesterol, while a decrease in HDL cholesterol levels (p < 0.001) was found (Supplementary Table 4).

## 4 Discussion

Our results indicate that, in the Geneva canton, 1) prevalence of dyslipidaemia and cholesterol levels are decreasing; 2) among people diagnosed with dyslipidaemia, treatment rates are decreasing; 3) among people treated for dyslipidaemia, controls rates are high, although the use of high potency statins is suboptimal, and 4) the issuing of the ESC or AHA guidelines had no effect on treatment or control levels, but was associated with a further decrease in total and LDL-cholesterol levels.

### 4.1 Trends in reported awareness, treatment and control rates

Over a 15 years study period, prevalence of dyslipidaemia decreased, awareness tended to increase, treatment among aware participants decreased, and control rates among treated participants tended to increase. Those findings are comparable to other studies conducted in France [19] and to a lesser degree in Lithuania, where the decrease was found only in men [20]. Conversely, they do not replicate previous findings from the Swiss health surveys, where self-reported prevalence of dyslipidemia increased [21].

Awareness tended to increase, although only for age-standardized rates. This trend is slightly similar to the one observed in the USA, where awareness rates increased until 2004 but remained stable afterwards [22].

Control rates tended to increase during the study period, although the increase was not statistically significant. In 2019, still between one-sixth (crude rates) and one-half (age-standardized rates) of treated participants did not achieve control. Those values are close to those reported in Portugal (50% control) [23], lower than in the USA (64% in 2009–2010) [22] and higher than in Spain (28% in 2005) [24] or China (7% control) [25]. Conversely, they are lower than reported in the Swiss health survey (75.1%), where the data was self-reported [21]. The reasons for the relatively low control level can be due to the use of differing thresholds/guidelines [26], low adherence to treatment by patients, or therapeutic inertia [27]. Overall, our results indicate that management of dyslipidaemia is perfectible in the canton of Geneva.

### 4.2 Factors associated with awareness, treatment and control

Women were less likely to present with dyslipidaemia, more likely to be aware of their status, less likely to receive treatment and more likely to be controlled. Similar findings were observed in China, except that women had a higher likelihood of being treated [25]. The lower treatment rates could be due to the false belief by doctors that dyslipidaemia is of a lesser concern in women [28], prompting the need for an adequate, gender-unbiased management of dyslipidaemia.

Elderly people were more likely to present with dyslipidaemia, less likely to be aware, more likely to be treated and less likely to be controlled, a finding also reported elsewhere [25]. The lower control rates could be due to increased number of medications prescribed (i.e. polymedication) among elderly people, thus reducing their compliance or leading to less powerful drugs to avoid interactions [29,30].

Higher educational level was associated with lower treatment rates, a finding also reported in another Swiss study [9], China [25] and Spain, where treatment rates in 2005 were 41% in subjects with elementary education vs. 31% in subjects with a university degree [24]. Possible explanations include shifting to non-allopathic medications [31,32], or different prescription strategies by GPs according to the health literacy of patients.

Swiss nationals presented with higher prevalence, lower awareness and higher treatment levels of dyslipidemia. A possible explanation for the higher prevalence levels is that many non-Swiss participants come from Southern Europe and consume a healthier diet, leading to lower cholesterol values [33]. Conversely, the higher treatment levels among Swiss nationals could be due to the high costs of health care in Switzerland, as non-Swiss participants tend to have a lower socio-economic status and might forgo health care for economic reasons [34,35].

Presence of other CVD risk factors such as smoking, high BMI, hypertension, diabetes and previous history of CVD were associated with a higher likelihood of being treated, suggesting that they are taken into consideration when prescribing a lipid-lowering drug treatment. Whether those risk factors are used to compute CVD risk remains to be assessed. Overall, our results indicate that efforts should be directed towards a better recognition of dyslipidaemia as a risk factor in women and a better management of this condition in the elderly. The inverse association between education and treatment should be further explored.

### 4.3 Trends in lipid-lowering drugs

Statin prescription decreased during the study period, a finding in contradiction with increasing trends observed in other countries [[36], [37], [38]]. Possible explanations include statin withdrawal due to negative news in the media [39] or their replacement by other lipid-lowering drugs, as observed in our study. Still, by the end on 2019, there was little information regarding the clinical efficiency of lipid-lowering drugs other than statins, and the reasons for such a decrease remain to be assessed.

Conversely, the decrease in statin prescription rates was accompanied by a shift from the least to the most potent statins. This shift has been observed in the USA [37], but to a lesser degree in France [36] or China [40]. A possible explanation would be the issuing of guidelines [41,42], although no association with the ESC guidelines of 2011 or 2016, or the AHA guidelines of 2013 were found. Other explanations could be the higher efficiency and better tolerance of the new generation statins [43].

### 4.4 Trends in lipid levels

Both total and LDL cholesterol levels decreased, a finding also observed for other European countries such as the Czech Republic [44], Germany [45] and Sweden [46], and also for the US population [47]. The reasons for such a decrease cannot be explained by higher prescription rates of lipid-lowering drugs, as the percentage of people diagnosed and treated decreased during the study period. Other factors should be considered, such as improvements in dietary intake [48] or physical activity [49]. This change in lifestyle could also explain the increase in HDL cholesterol levels observed. Our results suggest that cholesterol levels are decreasing in the Geneva population, and that this decrease might be more related to changes in lifestyle than in lipid-lowering drug prescription.

### 4.5 Effect of guidelines on treatment rates and lipid levels

No effect of the issuing of the ESC dyslipidaemia guidelines was found regarding treatment and control rates of dyslipidaemia. Our results do not confirm a previous systematic review, which reported that target achievement increased significantly over time after publication of the ESC 2011 guidelines [50] or a study conducted in the USA, where statin prescription increased after the publication of the AHA 2013 guidelines [5]. Conversely, the decrease in total and LDL cholesterol levels tended to accelerate after the issuing of the ESC guidelines. A possible explanation would be the prescription of more potent statins or the adoption of a healthier lifestyle as indicated previously [48,49].

### 4.6 Strengths and limitations

The main strength of this study is the use of the same questionnaire during the whole study period, together with standardized methods to assess CVD risk factors. This study is also based in successive random samples of the Geneva population collected using the same methodology, thus facilitating the assessment of trends.

This study also has several limitations. First, prevalence of dyslipidaemia was based on the ESC guidelines 2016, which might not be adequate for previous years; still, it allowed a standardization of the definition of dyslipidaemia, and other criteria such as awareness and treatment were used as in another study [15]. Second, the study was conducted in a single canton of Switzerland, and results might not be generalizable to the whole country, as differences in cardiovascular risk factor screening and management have been reported between administrative regions [51]. Still, they are in agreement with a similar study conducted in another Swiss city [9]. Third, risk categorization among treated participants was underestimated, as lipid levels used to estimate risk were decreased; hence, it is likely that control rates are overestimated. Fourth, the Bus Santé recruited only participants aged less than 75, and it would have been of interest to assess prevalence and management rates in participants older than 75 years. The ongoing CoLaus|PsyCoLaus study would allowing assessing those rates [9]. Finally, it was not possible to assess non-pharmacological interventions, or to detail the non-statin drugs, due to the small number of participants taking them.

## 5 Conclusion

In the canton of Geneva, only one-fifth of participants diagnosed with dyslipidaemia are treated, but most are adequately controlled. Women are undertreated but better controlled than men. Guidelines do not seem to influence significantly trends and the most potent lipid-lowering drugs are underused.

## Credit author statement

**Valeriya Chekanova**: Investigation, Methodology, Writing – Original draft preparation, Visualization. **Carlos de Mestral**: Reviewing and editing. **Pedro Marques-Vidal**: Conceptualization, Methodology, Data curation, Formal analysis, Writing – Original draft preparation, Visualization. **Idris Guessous**: Funding acquisition, Reviewing and editing. **Silvia Stringhini**: Reviewing and editing.

## Funding disclosure

(This work is funded by the 10.13039/501100006388Geneva University Hospitals through the General Directorate of Health 10.13039/501100011723Canton of Geneva) http://ge.ch/dares/sante/accueil.html. The funders had no role in study design, data collection and analysis, decision to publish, or preparation of the manuscript.

## Declaration of competing interest

The authors report no relationships that could be construed as a conflict of interest.
