# Supplementary material for: Trends and determinants of prevalence, awareness, treatment and control of dyslipidaemia in canton of Geneva, 2005–2019: Potent statins are underused
Source: Int J Cardiol Cardiovasc Risk Prev. 2023 May 19;18:200187. doi: 10.1016/j.ijcrp.2023.200187 (PMC10209490; doi:10.1016/j.ijcrp.2023.200187)
Supplement: Multimedia component 1 [file mmc1.pptx]

## Slide 1
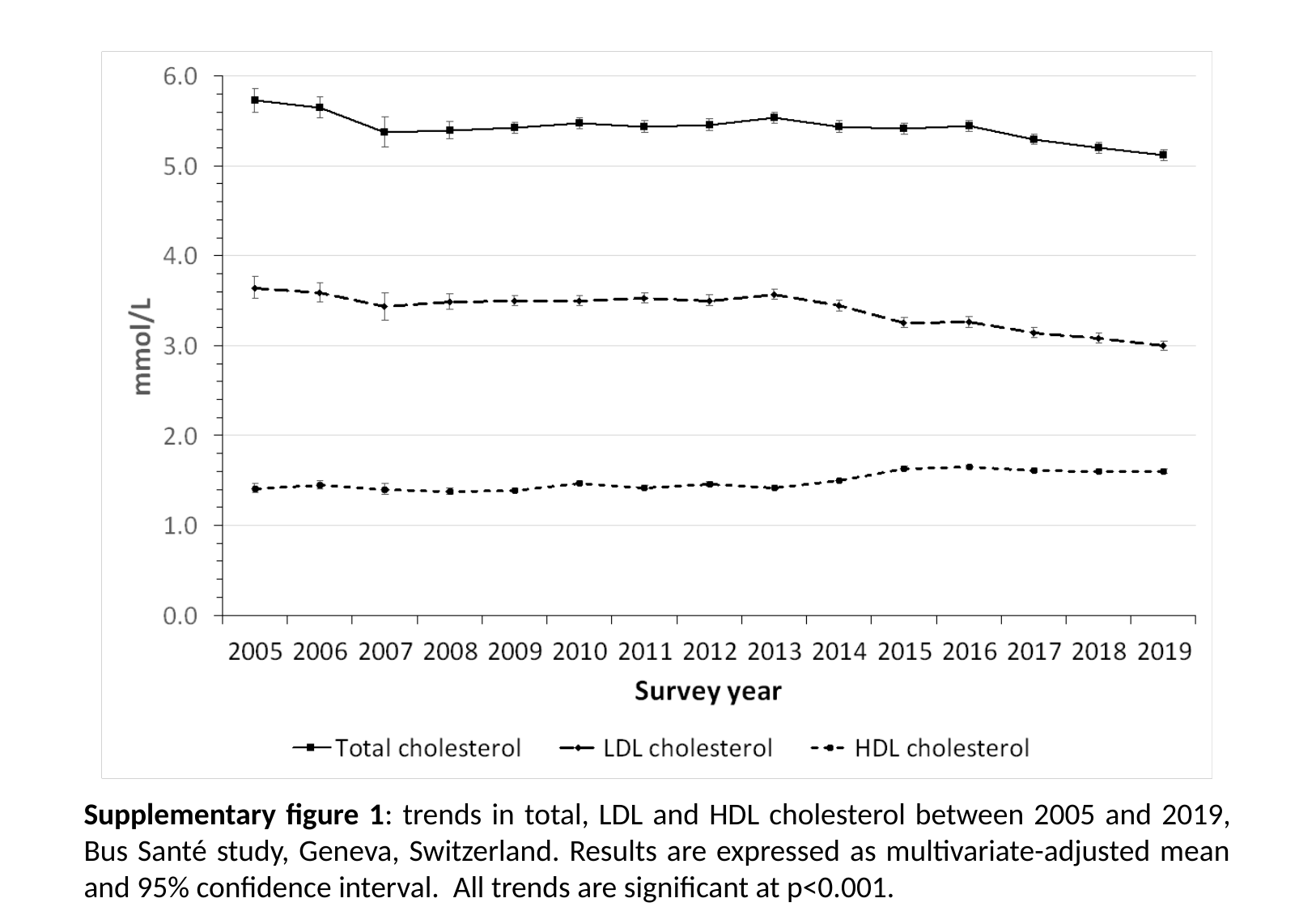

Supplementary figure 1: trends in total, LDL and HDL cholesterol between 2005 and 2019, Bus Santé study, Geneva, Switzerland. Results are expressed as multivariate-adjusted mean and 95% confidence interval. All trends are significant at p<0.001.
