# Supplementary material for: Trends and determinants of prevalence, awareness, treatment and control of dyslipidaemia in canton of Geneva, 2005–2019: Potent statins are underused
Source: Int J Cardiol Cardiovasc Risk Prev. 2023 May 19;18:200187. doi: 10.1016/j.ijcrp.2023.200187 (PMC10209490; doi:10.1016/j.ijcrp.2023.200187)
Supplement: Multimedia component 2 [file mmc2.docx]

**Supplementary information**

**Supplemental table 1:** statin classification regarding generation and potency.

| **Statin** | **ATC code** | **Generation** | **Potency** |
| --- | --- | --- | --- |
| lovastatin | C10AA02 | First | Low |
| pravastatin | C10AA03 | First | Low |
| fluvastatin | C10AA04 | First | Low |
| simvastatin | C10AA01 | Second | Intermediate |
| pitavastatin | C10AA08 | Second | Intermediate |
| atorvastatin | C10AA05 | Second | High |
| rosuvastatin | C10AA07 | Third | High |

Statins were ordered according to their potency. Adapted from {Maji, 2013 #86;Stone, 2014 #85}.

**Supplementary table 2**: thresholds to define dyslipidemia according to the SCORE equation recalibrated for Switzerland.

| **Risk (%)** | **Dyslipidemia** | **Control** |
| --- | --- | --- |
| ≥10, or very high risk | ≥1.8 | <1.8 |
| ≥5 to <10, or high-risk | ≥2.6 | <2.6 |
| ≥1 to <5 | ≥4.0 | <4.0 |
| <1 | ≥5.0 | <5.0 |

Values are for LDL-cholesterol and in mmol/L. Adapted from {Catapano, 2016 #60}.

**Supplementary table 3**: comparison between included and excluded participants, Bus Santé study, Geneva, Switzerland.

|  | **Included** | **Excluded** | **P-value** |
| --- | --- | --- | --- |
| N | 11,295 | 1820 |  |
| Woman (%) | 5847 (51.8) | 919 (50.5) | 0.314 |
| Age (years) | 50.7 ± 11.9 | 37.1 ± 30.1 | <0.001 |
| Educational level (%) |  |  | <0.001 |
| Primary | 940 (8.3) | 103 (6.2) |  |
| Secondary | 4919 (43.6) | 802 (48.1) |  |
| Tertiary | 5436 (48.1) | 762 (45.7) |  |
| Swiss nationality (%) | 7470 (66.1) | 1289 (70.5) | <0.001 |
| Marital status (%) |  |  | <0.001 |
| Single | 1376 (12.2) | 875 (48.0) |  |
| Married/couple | 7717 (68.3) | 775 (42.5) |  |
| Divorced | 1620 (14.3) | 128 (7.0) |  |
| Widowed | 582 (5.2) | 44 (2.4) |  |
| Smoking categories (%) |  |  | <0.001 |
| Never | 5414 (47.9) | 955 (54.2) |  |
| Former | 3516 (31.1) | 348 (19.8) |  |
| Current | 2365 (20.9) | 459 (26.1) |  |
| Body mass index (kg/m^2^) | 25.2 ± 4.5 | 23.9 ± 4.3 | <0.001 |
| Body mass index categories (%) |  |  | <0.001 |
| Normal | 6018 (53.3) | 1058 (68.4) |  |
| Overweight | 3760 (33.3) | 352 (22.8) |  |
| Obese | 1517 (13.4) | 136 (8.8) |  |
| Hypertension (%) | 2669 (23.6) | 204 (11.2) | <0.001 |
| Diabetes (%) | 828 (7.3) | 63 (3.5) | <0.001 |
| History of CVD (%) | 501 (4.4) | 47 (2.6) | <0.001 |

Results are expressed as number of participants (column %) for categorical variables and as average ± standard deviation for continuous variables. Between-groups comparisons performed using chi-square for categorical variables and student’s t-test for continuous variables.

**Supplementary table 4**: trends in total, HDL and LDL cholesterol before and after the issuing of the ESC dyslipidemia guidelines of 2011 and 2016 and of the AHA guidelines of 2013, Bus Santé study, Geneva, Switzerland.

|  | **Total cholesterol** | **HDL cholesterol** | **LDL cholesterol** |
| --- | --- | --- | --- |
| ESC 2011 guidelines |  |  |  |
| Before | -0.032 (-0.051 ; -0.014) | 0.006 (0 ; 0.012) | -0.011 (-0.028 ; 0.005) |
| After | -0.053 (-0.063 ; -0.043) | 0.025 (0.021 ; 0.029) | -0.082 (-0.091 ; -0.073) |
| Test for interaction | 0.040 | <0.001 | <0.001 |
| AHA 2013 guidelines |  |  |  |
| Before | -0.009 (-0.021 ; 0.004) | 0.005 (0.001 ; 0.010) | -0.001 (-0.012 ; 0.010) |
| After | -0.067 (-0.082 ; -0.053) | 0.009 (0.003 ; 0.015) | -0.08 (-0.094 ; -0.067) |
| Test for interaction | <0.001 | 0.231 | <0.001 |
| ESC 2016 guidelines |  |  |  |
| Before | -0.008 (-0.016 ; -0.001) | 0.025 (0.022 ; 0.028) | -0.028 (-0.035 ; -0.022) |
| After | -0.086 (-0.127 ; -0.046) | -0.006 (-0.023 ; 0.011) | -0.070 (-0.107 ; -0.033) |
| Test for interaction | <0.001 | <0.001 | 0.027 |

Results are expressed as slope and (95% confidence interval) for a one-year increase obtained using linear regression stratified on period and adjusting for age (continuous), gender (man, woman), nationality (Swiss/non-Swiss), marital status (4 categories), smoking categories (never, former, current), BMI categories (normal, overweight, obese), hypertension (yes/no), diabetes (yes/no) and personal history of CVD (yes/no). Interaction assessed using ANOVA adjusting for the same covariates.
